# Long Non-Coding RNAs as Master Regulators in Cardiovascular Diseases

**DOI:** 10.3390/ijms161023651

**Published:** 2015-10-05

**Authors:** Krystal Archer, Zuzana Broskova, Ahmed S. Bayoumi, Jian-peng Teoh, Alec Davila, Yaoliang Tang, Huabo Su, Il-man Kim

**Affiliations:** 1 Department of Medicine, Medical College of Georgia, Georgia Regents University, Augusta, GA 30912, USA; E-Mails: karcher@gru.edu (K.A.); adavila@gru.edu (A.D.); yaotang@gru.edu (Y.T.); 2 Vascular Biology Center, Medical College of Georgia, Georgia Regents University, Augusta, GA 30912, USA; E-Mails: zbroskova@gru.edu (Z.B.); abayoumi@gru.edu (A.S.B.); jteoh@gru.edu (J.-p.T.); hsu@gru.edu (H.S.); 3 Department of Biochemistry and Molecular Biology, Medical College of Georgia, Georgia Regents University, Augusta, GA 30912, USA

**Keywords:** chromatin, epigenetic regulation, gene regulation, heart disease, non-coding RNAs

## Abstract

Cardiovascular disease is the leading cause of death in the United States, accounting for nearly one in every seven deaths. Over the last decade, various targeted therapeutics have been introduced, but there has been no corresponding improvement in patient survival. Since the mortality rate of cardiovascular disease has not been significantly decreased, efforts have been made to understand the link between heart disease and novel therapeutic targets such as non-coding RNAs. Among multiple non-coding RNAs, long non-coding RNA (lncRNA) has emerged as a novel therapeutic in cardiovascular medicine. LncRNAs are endogenous RNAs that contain over 200 nucleotides and regulate gene expression. Recent studies suggest critical roles of lncRNAs in modulating the initiation and progression of cardiovascular diseases. For example, aberrant lncRNA expression has been associated with the pathogenesis of ischemic heart failure. In this article, we present a synopsis of recent discoveries that link the roles and molecular interactions of lncRNAs to cardiovascular diseases. Moreover, we describe the prevalence of circulating lncRNAs and assess their potential utilities as biomarkers for diagnosis and prognosis of heart disease.

## 1. Introduction

Since the discovery of the human genome, technological advances have enabled scientists to study the whole genome in detail and its implication in physiology and pathology. In the early 1960s, it was estimated that the human genome consisted of at least two million of protein-coding genes. However, the launching of the Human Genome Project in 1990 dropped this number to 20,500 of coded genes, which is only about 2% of the genome [1]. The other 98% consisted of non-coding genes and was deemed as “transcriptional noises” or “genetic junks” due to the assumption that they played no necessary cellular functions. Therefore, researchers have ignored these non-coding regions, but non-coding RNAs (ncRNAs) transcribed from these regions are recently emerging as important regulators of cellular processes with many implications in human diseases [2].

Unlike messenger RNAs (mRNAs), ncRNAs do not translate to generate proteins. Non-coding DNA sequences are actively transcribed into ncRNA sequences, which include microRNA (miRNAs or miRs), short interfering RNAs (siRNAs), and long non-coding RNAs (lncRNAs) [3]. MiRs are short ncRNA sequences with a length of 20–22 base pairs and have important regulatory functions in health and disease. In mammalian species, miRs post-transcriptionally regulate gene expression by inhibiting protein synthesis or promoting mRNA degradation through the binding to specific complementary mRNA sequences mainly in the 3′-untranslated region (3′-UTR) [4]. SiRNAs comprise of 20–25 base pairs in length and regulate gene expression, mostly through the RNA interference (RNAi) pathway [5]. According ncRNA classification, lncRNAs are more than 200 base pairs in length. They are transcribed by RNA polymerase II, polyadenylated, and subjected to splicing. LncRNAs also have promoter structures and are pervasively expressed across the genome [6].

Recently, lncRNAs have emerged as important players in regulating gene expression. For example, they can regulate neighboring genes in cis-formation. They can also function in trans-acting formation to regulate the expression of genes, which are not located closely with lncRNAs. LncRNAs play important roles in the initiation and development of diseases, particularly cardiovascular diseases [7]. They can control the expression of disease-related genes by functioning as molecular scaffolds to regulate chromatin modifications and influencing the epigenetic programs of the transcriptome [8]. Moreover, lncRNAs are actively involved in biological processes including cardioprotection and they can act as endogenous miR sponges by interacting with miRs to control gene expression [9,10]. For instance, it is shown that lncRNAs, such as Braveheart (Bvht), elicit gene activation or suppression to stimulate stem cell differentiation during cardiac development [9]. Another lncRNA cardiac hypertrophy-related factor (Chrf) was shown to sequester miR-489 to inhibit its action of targeting *myeloid differentiation primary response gene 88* (*Myd88*), which induces cardiac hypertrophy through the NF-κB system [10].

In the following sections, we describe classes of lncRNAs, genetic functions of lncRNAs and their roles in cardiovascular development and diseases, followed by a summary of recent findings that utilize lncRNAs as diagnostic markers of heart disease.

## 2. Representative Classes of LncRNAs

**Figure 1:**
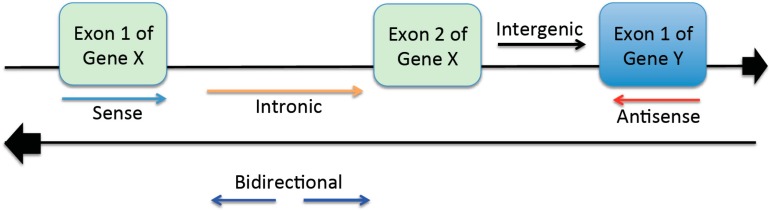
Schematic diagram of representative classes of lncRNAs. Long non-coding RNAs are divided into five subgroups. The groups are based on the genomic locations where the lncRNAs are transcribed from the non-coding sequences.

LncRNAs are classified into five subclasses, which include intergenic, intronic, sense overlapping, anti-sense, and bidirectional lncRNAs. Each subclass is categorized by the genomic location of lncRNAs in relation to their neighboring encoding regions (Figure 1). Long intergenic non-coding RNAs (lincRNAs) are transcribed by segments of DNA located between genes [11]. Historically, lincRNAs have been referred to as “junk genes” because they were thought to have no functional significance [12]. However, recent studies have revealed that lincRNAs may control genes in close proximity to their locations by modulating promoters or enhancers of the genes [13]. Intronic lncRNAs are transcribed by sequences of DNA that intercept a gene sequence. They are thought to modulate gene expression through various transcriptional mechanisms. Both lincRNAs and intronic lncRNAs have poly (A) tails and influence various processes in health and disease [14,15]. Sense lncRNAs are transcribed from a sense strand of protein-coding genes that contain exons. They can overlap or cover protein-coding genes. In contrast, anti-sense lncRNAs are formed from the transcription of anti-sense protein-coding genes [16,17]. Bidirectional lncRNAs are spaced approximately 1 kb apart and transcribed in opposite directions of each other (Figure 1). It has been shown that they have similar functions as their protein-coding counterparts [18].

LncRNAs transcribed from antisense protein-coding genes or sense protein-coding genes can be retrieved by cap-analysis gene expression (CAGE) and oligo-dT guided reverse transcription, indicating similarities with mRNAs such as 5′-capping and 3′-polyadenylation [19]. Although it has been suggested that lncRNAs possess no protein-coding potential, some lncRNAs can utilize their own protein-coding sequences. For example, steroid receptor RNA activator (SRA1) translates a protein and functions as a scaffold by using its RNA sequence to form a complex with repressor proteins or co-activators [20]. Also, early nodulin 40 (ENOD40) is suggested to have translational potential and functions as a master regulator in subcellular localization in plants [21]. It is anticipated that lncRNA classification will be continuously changed as the field of lncRNAs is expanded and new annotated lncRNAs are identified.

## 3. Global Mechanisms of LncRNA Functions

**Figure 2:**
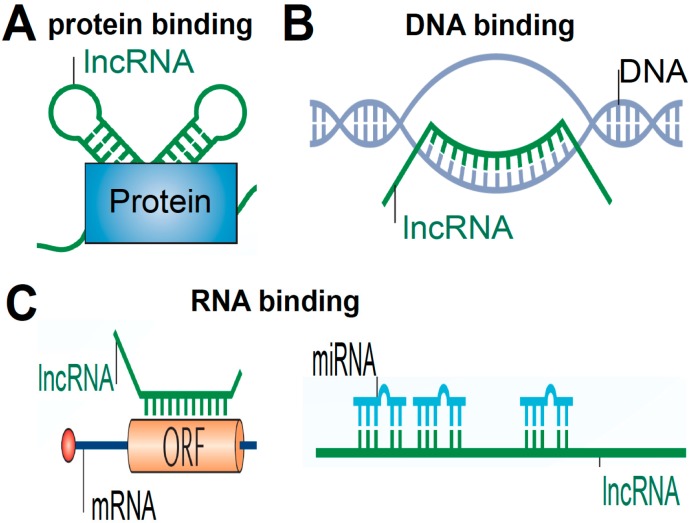
Schematic diagram of lncRNA mechanisms of action. LncRNAs function to regulate gene expression by diverse mechanisms. (**A**) LncRNAs act as molecular guides and scaffolds to interact with protein complexes. LncRNAs also act as molecular decoys for proteins including transcription factors; (**B**) lncRNAs modulate the epigenetics through guiding chromatin-modifying complexes to target genomic DNA loci; (**C**) lncRNAs act as endogenous sponges for other RNAs such as mRNAs and miRNAs. ORF: open reading frame.

It has been shown that lncRNAs can elicit gene activation or suppression by interacting with proteins, DNAs and RNAs including miRNAs [2,3,6,7,8]. LncRNAs can act as molecular scaffolds to affect gene expression by interacting with proteins such as transcription factors or components of chromatin modifying complexes (Figure 2A). Other proposed mechanisms of action of lncRNAs include targeting proteins to specific genomic sites such as promoter regions by complementary interactions with DNAs. An alternative scenario is that interactions with DNAs prevent the binding of specific proteins to the DNA regions (Figure 2B). Lastly, lncRNAs can also base pair with other RNA molecules such as mRNAs or may act as endogenous sponges for miRNAs (Figure 2C), which in turn negatively regulates other RNAs’ functions [2,3,6,7,8].

As aforementioned, lncRNAs exhibit several molecular functions such as signaling, decoys, guides, and scaffolds to regulate health and disease [2,22]. In the last decade, it has been found that lncRNAs participate in molecular signaling by being transcribed at specific spatial and temporal points. It has been also shown that lncRNAs can act as signaling molecules to modulate gene or allele function. For instance, lncRNAs have been shown to involve in X chromosome inactivation [22,23]. Mammalian organisms are diploid and are born with two alleles per an autosomal gene. In female eggs, two X chromosomes are present but only one allele can be expressed within the egg. Xist, a lncRNA, regulates gene expression by interacting with one of the X chromosomes and inactivating X alleles [23]. This process occurs during female development in which Xist RNA is produced from the inactivated X allele. Xist interacts with the X allele from which it was originally transcribed. The binding of Xist to the inactivated X allele represses the allele’s overall capability of gene expression. Xist can be repressed by an antisense lncRNA called Tsix, while it can also be activated by another lncRNA called Jpx [22]. The presence of the transcribed Xist from X allele demonstrates how active genomic silencing occurs through molecular signaling and the negative regulation of lncRNAs [23].

LncRNAs act as negative regulators to down-regulate the functions of targeted proteins [24]. For example, lncRNAs act as molecular decoys and regulate cellular processes by targeting and sequestering specific proteins. An lncRNA named PANDA was revealed to have decoy functions by which it deactivated nuclear transcription factor Y subunit α (NF-YA), a positive regulator of apoptosis. During low levels of DNA damage in the cell, PANDA becomes activated and is transcribed in a p53-dependent manner. PANDA then directly binds to NF-YA to protect the cells from undergoing apoptosis. It is also shown that knockdown of PANDA in human fetal fibroblasts led to increased levels of NF-YA and cell apoptosis [24].

LncRNAs can also function as molecular guides by directing proteins to specific targets in the cell and determining gene expression in the *cis* and *trans* positions [25]. A central platform used as a molecular scaffold is another function of lncRNAs. For instance, lncRNAs are considered as important elements for cellular stabilization [26]. One of the best examples is the regulation of telomerase by lncRNAs. Telomerase is a crucial molecular protein that functions to add back telomeric DNA repeats that are removed from the ends of chromosomes. The components of the telomerase enzyme include an integral RNA subunit called telomerase RNA component (TERC) and a catalytic protein subunit called telomerase reverse transcriptase (TERT). TERC serves as a template for repeat synthesis and provides important binding activity [27]. LncRNAs, which influence the stability of telomerase, have been found to affect the template usage [26,28]. LncRNAs can be also involved in the reassembling of the telomerase complex as well as RNA motifs [26,29].

## 4. Identification of LncRNAs as Major Regulators in Human Disease

LncRNAs have been implicated in the pathogenesis of disease by interacting with non-coding and coding genes to regulate a diverse network of cellular processes [30]. For example, an lncRNA named cardiac apoptosis-related lncRNA (CARL) inhibits miR-539 by acting as an endogenous sponge of miR-539. Because miR-539 induces mitochondrial fission and apoptosis in the heart by targeting the gene *PHB2* and downregulating its expression, CARL was shown to increase *PHB2* gene expression and inhibit mitochondrial fission or apoptosis in cardiomyocytes [31]. In a study conducted by Shirasawa *et al.*, the T allele of Ex9b-single-nucleotide polymorphism (SNP)10 in a lncRNA was discovered to increase the risk of an individual for autoimmune thyroid disease, which is caused by abnormal immune responses [32]. Ex9b-SNP10 was found in the intron 9 of *zinc finger and AT hook domain containing gene* (*ZFAT*) and an antisense lncRNA called small antisense transcript of ZFAT (SAS-ZFAT) is located in the promoter region. It was found that in the presence of Ex9b-SNP10, SAS-ZFAT was upregulated and inhibited its sense counterpart. The significance of the study is that that SAS-ZFAT was only found in CD19+ B cells, indicating that SAS-ZFAT is a major regulator of B cell function and susceptibility to autoimmune thyroid disease [32].

Chromatin-modifying proteins like Sin3A and coREST are being used to target stroke-induced lncRNAs following ischemic stroke. A recent study showed that the levels of lncRNAs were increased after transient focal ischemia. It was shown that 177 of a pool of 2497 lncRNAs bind to chromatin-modifying proteins. Of the 177, only 26 were bound to Sin3A while 11 were associated with coREST following ischemic attack. Further analysis revealed that these lncRNAs share common locus and may interact with each other through an epigenetic signaling [33].

Among multiple human diseases, we focus on lncRNAs’ roles in cardiovascular development and disease in the following sections.

## 5. LncRNAs in Cardiovascular Development

It is becoming more evident that lncRNAs play important roles in cardiovascular development and embryogenesis [34,35]. In fact, lncRNAs regulate the processes that sustain cell transformation and organogenesis. During organogenesis, lncRNAs regulate genes and miRs that are activated during cell fate and cell maintenance to ensure a proper development and cellular transformation [35]. LncRNAs are essential components of the regulation of genes in embryonic stem cells because alteration of gene expression may cause an imbalance in the maintenance of stem cell differentiation [36]. For example, the cellular pluripotency is correlated with the expression of lncRNAs that are regulated by transcription factors such as Nanog and octamer-binding transcription factor 4 (Oct4) [37].

**Figure 3:**
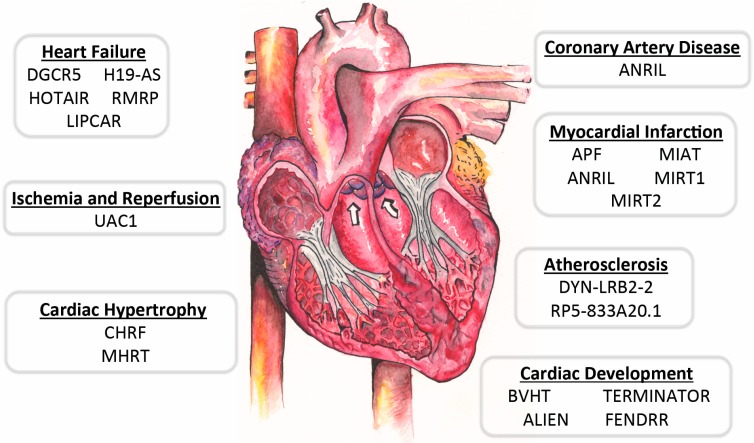
Roles of lncRNAs in cardiovascular development and disease. Overview of lncRNAs discussed in this review is shown. The white arrows show the blood flow.

LncRNAs also play crucial roles in mammalian heart development and cellular lineage. In a study conducted in mice, Bvht was found to participate in cardiovascular-associated cell differentiation. The study revealed that Bvht was required for the development of nascent mesoderm, which influenced cardiac growth and activated a network of genes associated with cardiac differentiation. Also, Bvht was involved in cardiomyocyte differentiation and was shown to modulate cell fate in neonatal cardiomyocytes (Table 1 and Figure 3) [9].

**Table 1:** LncRNAs associated with cardiovascular development and diseases.

Disease	LncRNA Name	Effect *	Reference
Atherosclerosis	CDKNA/B	↑	[38]
	DYN-LRB2-2	↑	[39]
	RP5-833A20.1	↑	[40]
Cardiac Hypertrophy	CHRF	↑	[10]
	MHRT	↑	[41]
Heart Failure	LIPCAR	↓/↑	[42]
Coronary Artery Disease	ANRIL	Unknown	[43]
Ischemia and Reperfusion	UAC1	↓	[44]
Cardiac Development	BVHT	↑	[9]
	TERMINATOR	↓	[45]
	ALIEN	Unknown	[45]
	AK143260	Unknown	[45]
	FENDRR	Unknown	[46]
Myocardial Infarction	MIAT	Unknown	[47]
	APF	↑	[48]
	ANRIL	Unknown	[43]
	MIRT1	↑	[49]
	MIRT2	↑	[49]

* **↑**: up-regulation; **↓**: down-regulation.

It has been also shown that lncRNAs interact with coded genes to regulate cellular lineage. During stem cell differentiation, cells lacking lncRNA AK143260 do not differentiate into specific cardiac cells. AK143260 is important for the activation of a gene network which is involved in the transition of cardiac mesoderm into multipotent cardiac cell progenitors [45]. Moreover, lncRNA TERMINATOR controls the identity of pluripotent stem cells and alters the function of lncRNA ALIEN, which is related to cardiovascular development (Table 1 and Figure 3) [45]. The expression of lncRNA PUNISHER compromises the functions of cardiac endothelial cells and lncRNA Bvht controls *MesP1* gene, which is a master regulator of genes that modulate the differentiation of cardiac cells in vertebrates [45,50]. It has been also demonstrated that lncRNA Bvht interacts with the polycomb repressive complex 2 (PRC2), which has histone methyl-transferase activity [51]. The PRC2 modulates *MesP1* and other important genes for cellular commitment and heart organogenesis [50]. Lastly, lncRNA Fendrr has been shown to be responsible for the development of heart walls [46]. The study from transgenic animal models lacking Fendrr showed that Fendrr controls important transcription factors such as GATA binding protein 6 (GATA-6), NK2 homeobox 5 (NKX2.5), forkhead box f1 (FOXF1), T-box 3 (TBX3), iroquois homeobox 3 (IRX3), and paired-like homeodomain 2 (PITX2) during the heart organogenesis (Table 1 and Figure 3) [46].

## 6. LncRNAs and Cardiovascular Disease

Using high-throughput sequencing techniques such as RNA sequencing and annotation of chromatin-state maps, lncRNAs have emerged as master regulators of heart disease in humans and many animal models [51,52]. Recent studies have revealed that lncRNAs are dysregulated during various pathological conditions, making them the ideal candidates for therapeutic targets and biomarkers [42]. LncRNAs such as Bvrt, Fendrr, ANRIL, MIAT, and MyHeart (Mhrt) play important roles in cardiovascular development and heart disease, including myocardial infarction, cardiomyopathy, heart failure, and atherosclerosis [9,41,43,46,47,53]. For instance, lncRNA Mhrt regulates BRG1 to prevent heart failure [41,53]. Over the past decade, multiple studies have associated lncRNAs with various forms of cardiovascular diseases (Table 1 and Figure 3). With the increasing mortality of heart diseases in the United States, efforts have been directed to discover new connection between lncRNAs and cardiovascular diseases in the hope for novel therapies. Here, we summarize major discoveries in lncRNAs associated with various cardiovascular diseases.

### 6.1. Roles of LncRNAs in Myocardial Infarction

Sudden cardiac death accounts for approximately 50% of all heart-related death in the United States. Coronary artery disease is the most prevalent cause of sudden cardiac death and is commonly followed by acute myocardial infarction (AMI) if not treated [54]. Coronary artery disease accounts for 22% of early deaths and 15% of late deaths in individuals with cardiovascular disease [55]. It is known that coronary artery disease is featured by the transcriptional reprogramming, which activates a network of cardiac signals that interact and converge on cardiac transcription factors such as serum response factor (SRF), NKX2.5, myocyte-specific enhancer factor 2C (MEF2C), GATA binding protein 4 (GATA4), and TBX3. These transcriptional factors collectively activate specific temporal and spatial programs to generate cardiac pathological remodeling that is controlled by lncRNAs. For example, conditions such as anoxia/re-oxygenation (A/R) and ischemia/reperfusion (I/R) increase the transcription of lncRNA autophagy-promoting factor (APF) [48]. In a study conducted by Wang K. *et al*., APF was discovered to exacerbate myocardial infarction (MI) injury by interacting with miR-188-3p. MiR-188-3p suppresses the expression of ATG7, a key regulator of autophagy that is known to be crucial in regulating cell death and survival in MI [48]. Multiple lines of evidence were provided to prove that APF modulates MI injury through the inhibition of miR-188-3p. For instance, ATG7 was significantly decreased in the presence of miR-188-3p and knockdown of miR-188-3p resulted in increased levels of endogenous ATG7 expression. However, knockdown of APF in cardiomyocytes showed decreased ATG7 expression. Overall, these data suggest that miR-188-3p is indeed a target of APF and that both miR-188-3p and APF may play important roles in the modulation of ATG7 levels during MI [48]. Furthermore, a microarray-based study showed that MI is associated with the upregulation of MI-associated transcript 1 and 2 (MIRT1 and MIRT2) [49]. An lncRNA MI-associated transcript (MIAT) was also identified as a novel non-coding RNA that regulates MI. LncRNA MIAT is involved in pathological angiogenesis and is suggested as a predictor of MI because MIAT was measured in peripheral blood cells and proposed as a significant invariable predictor of MI (Table 1 and Figure 3) [47].

Recent studies have utilized an advanced technology called SNP Array to identify disease-associated SNPs on a genome-wide scale [47,56]. In a study used 52,608 haplotype-base SNP markers from 3435 MI patients and 3774 controls, several SNPs were linked to non-coding regions of the genome, which produced no translational protein product *in vitro* [47]. The findings of the study revealed six SNPs on chromosome 22q12.1 that had significant association with MI. Within the locus, authors identified a lncRNA MIAT, which has five exons and is 10 kb in length [47]. Overall, the study suggests that the change in MIAT expression by the SNPs is involved in the pathogenesis of MI. Additionally, genome-wide association studies have revealed regions on chromosome 9p that are associated with coronary artery disease [57]. An antisense lncRNA called ANRIL has been found in the LNK4 locus and several SNPs in the ANRIL locus on chromosome 9p are involved in coronary artery disease and diabetes (Table 1 and Figure 3) [43]. Several studies also have linked ANRIL in increasing the risks of intracranial aneurysm [58], breast cancer [59], glioma [60], and basal cell carcinoma [61].

Further studies have also proven an association between MI and lncRNAs. In a microarray-based study, it was found that 20 lncRNAs were up-regulated and 10 lncRNAs were down-regulated following MI in mice. It was noted that among the 30 lncRNAs, two specific lncRNAs, MIRT1 and MIRT2, were significantly up-regulated by 5-fold and 13-fold, respectively. MIRT1 and MIRT2 were associated with genes involved in left ventricular remodeling [49].

In a study conducted by Kumarswamy *et al.*, a global transcriptomic technology was used to measure plasma RNAs in patients experiencing heart remodeling following MI and in healthy individuals. The analyses showed that a mitochondrial lncRNA uc022bqs.1 (LIPCAR) was downregulated during the initial stages of MI, whereas it was upregulated during the later stages. Among a group of 344 individuals with systolic heart failure, patients with chronic heart failure had elevated levels of LIPCAR as compared to patients with continuous heart remodeling at one year after MI. Overall, the study showed that LIPCAR levels were indeed associated with future cardiovascular health (Table 1 and Figure 3) [42].

### 6.2. Roles of LncRNAs in Ischemia and Reperfusion Injury

LncRNAs were also studied in the pathology of cardiac ischemia/reperfusion (I/R). Liu *et al.*, analyzed the expression levels of lncRNAs in the early stage of reperfusion in the mouse infarct region. After ischemia, 64 lncRNAs were up-regulated while 87 lncRNAs were down-regulated among total 31,423 lncRNAs. A down-regulated lncRNA UAC1 played a pro-apoptotic role in primary cardiomyocytes by stimulating p27 protein levels, suggesting its role in I/R injury. This data indicated that aberrant lncRNA expression in the infarct region might cause imbalance in cell recovery and tissue necrosis (Table 1 and Figure 3) [44].

### 6.3. Roles of LncRNAs in Cardiac Hypertrophy and Heart Failure

Cardiac hypertrophy is the thickening of the myocardium and the subsequent heart failure, which is caused by a maladaptive response of the heart, is a common heart disease in the United States. Cardiac hypertrophy is associated with pressure or volume overload in the heart during various heart diseases such as hypertension and valve stenosis. In the initial phase of cardiac hypertrophy, the heart muscle begins to thicken, thus decreasing the chamber size and cardiac output. Cardiomyocytes then attempt to compensate for the decreased cardiac output by increasing their sizes. The imbalance between nutrient supply and demand in cardiomyocytes eventually leads to heart failure [3]. In 2013, over five million individuals had heart failure in the United States, prompting an urgent need for therapeutic and preventative solutions [62].

Clusters of lncRNAs have been associated with cardiac hypertrophy [9,42,44]. In pathological hypertrophy, Han *et al.*, identified a cluster of lncRNAs transcribed from *Myh7* loci and demonstrated a new mechanism for heart failure (lncRNA-chromatin mechanism) [41]. *Myh7* undergoes antisense transcription to produce a series of lncRNAs. Among them, an lncRNA called myosin heavy chain-associated RNA transcripts (Myheart or Mhrt) has been shown to be cardiac-specific and abundant in adult hearts. During heart failure, the Brg1-Hdac-Parp chromatin repressor complex prevents Mhrt transcription, which is essential for development of cardiomyopathy [41,53]. Mhrt has been found to protect the heart from cardiac stress by binding to the helicase domain of Brgl and thus decreasing Brg1’s association with its target gene, which results in the suppression of cardiac hypertrophy [41]. However, increased levels of stress stimuli have been found to down-regulate Mhrt through the activation of Brgl–Hdac–Parp chromatin complex, which exacerbates cardiac hypertrophy. Also, Mhrt inhibits Brg1 that is a chromatin-remodeling factor stimulated by stress and increases the levels of detrimental genes involved in cardiomyopathy. Because the human heart is also known to express Mhrt that is originated from *Myh7* loci, it is suggested that Mhrt has its potentially conserved biological function [41].

Another study by Liu *et al.*, sought to discover specific lncRNAs that may be important in heart failure. The study used myocardial-targeted pdk1 knockout (KO) mice on postnatal days 8 and 40. It was shown that 2024 lncRNAs or 4059 lncRNAs were dysregulated at postnatal day 8 or 40, respectively in the KO mice compared to control groups [44]. In addition, using advanced bioinformatic technologies, mitogen-activated protein kinase (MAPK) signaling pathway was found to be a major regulator in heart failure progression. In heart failure patients, four LncRNAs (DGCR5, H19-AS, HOTAIR, and RMRP) in plasma were differentially regulated compared to healthy people (Table 1 and Figure 3). Further analysis revealed that a sense overlap lncRNA called mkk7 was involved in heart failure. The lncRNA mkk7 was downregulated in cardiomyocytes isolated from mice on postnatal day eight in the pdk1 KO mice as compared to the control group. Overall, the study demonstrated a correlation between mkk7 levels and heart failure progression [44].

### 6.4. Roles of LncRNAs in Atherosclerosis

Atherosclerosis causes the narrowing of blood vessels due to immunological responses, which produce plaques in subendothelial spaces [63]. The formation of plaques is stimulated by monocytes that differentiate into macrophages [64]. The obstruction of blood vessels is considered as a high risk factor for atherosclerosis because it may cause high blood pressure and stroke [65,66].

Recently, some lncRNAs have been implicated in the etiology of plaque formation leading to atherosclerosis. In a study conducted by Hu *et al.*, a lncRNA called RP5-833A20.1 was found to regulate a gene called *nuclear factor IA* (*NFIA*) [40]. NFIA is a member of the nuclear factor I family which regulates gliogenesis [67]. NFIA regulates transcription by directly binding to DNA containing a TTGGC motif [68]. It was suggested that NFIA is a primary regulator in adipocyte formation and lipid development. Moreover, a study has revealed that aberrant expression of NFIA caused an increase in lipid droplet formation [69]. Another study showed that the lncRNA RP5-833A20.1 downregulates NFIA expression by inducing miR-382-5p expression, which leads to the formation of THP-1 macrophage-derived foam cells. The study noted that normal levels of NFIA suppress atherosclerotic plaque formation, while aberrant levels of NFIA expression leads to higher levels of plaque formation [40].

Reverse cholesterol transport is a physiological process to remove and mobilize lipids and cholesterols that might contribute to the plaque formation. Within this pathway, a transmembrane transport protein called ABCA1 functions to transport cellular cholesterols to its corresponding apolipoproteins [39,70]. In a mouse study, oxidized low-density lipoproteins (LDLs) stimulated an lncRNA called DYN-LRB2-2 (Table 1 and Figure 3), which promoted ABCA1-mediated cholesterol efflux and up-regulated G protein-coupled receptor 119 (GPR119). GPR119 was shown to be a key player in glucose and lipid metabolism, which regulates atherosclerotic plaque formation by decreasing cellular cholesterol levels and inflammation [39].

### 6.5. Abnormal Regulation of LncRNAs in Veins

Over a quarter of Americans are affected by varicose veins, which are enlarged and twisted veins due to obesity and aging [71]. LncRNAs have been also shown to regulate this disease. To identify aberrantly expressed lncRNAs in great saphenous veins, a study used 33,045 lncRNAs along with 30,215 mRNA probes and discovered that 557 lncRNAs and 950 mRNAs were significantly up-regulated between samples from 32 patients experiencing varicose vein excision and controls. Six lncRNAs (AF119885, AK021444, NR_027830, G36810, NR_027927, uc.345-) were further validated using quantitative real-time PCR. Four of them were associated with 11 mRNAs related in metabolic pathways as follows: LncRNA AF119885 was found to be related to the mRNAs CHAT and TMEM38B, lncRNA G36810 was found to be related to the mRNAs CCNO, EPC2, FAM13C and SHOC2, lncRNA NR_027927 was found to be related to the mRNAs EMX1 and SMC3, and lncRNA uc.345- was found to be related to the mRNAs ATXN7, HOXC4, and RTCD1. This study provides evidence that lncRNAs play important roles in great varicose saphenous vein disease [72].

## 7. Circulating LncRNAs as Novel Biomarkers for Heart Disease

Circulating lncRNAs have been reported as promising biomarkers for heart disease. In a microarray-based study, Li *et al.*, analyzed the expression levels of lncRNAs in whole blood, tissue, and plasma in mouse models. The study revealed that in an acute heart failure mouse model, 518 lncRNAs were upregulated while 908 were downregulated in the heart [73]. Moreover, a significant change in gene expression was observed in heart tissue in comparison to whole blood or plasma. The authors concluded that lncRNAs have strong potentials as biomarkers in cardiovascular diseases [73]. Other lncRNAs have been reported to have implications as biomarkers in heart disease. These include ANRIL and CDKN2A/B [38], which is associated with atherosclerosis, and MIAT [47] and LIPCAR [42], which play significant roles in MI and heart failure (Table 1 and Figure 3). As miRs have been identified as cardiac biomarkers, it is expected that the utility of lncRNAs as diagnostic markers of heart disease will be increased.

## 8. Conclusions

Once considered “genomic junk”, non-coding regions of the genome are shown to produce molecular lncRNAs which play critical roles in the regulation of cardiovascular diseases. Although several breakthroughs in understanding the mechanisms and roles of lncRNAs have occurred, researchers are still working to identify novel lncRNAs and their association with other molecules using bioinformatics and high-throughput sequencing technologies. Future research on the field of lncRNAs will ultimately unveil their utility as novel diagnostic and therapeutic strategies in cardiovascular medicine. However, many gaps still exist. For example, few large animal and human studies have been done. To completely translate existing basic research outcomes into clinical therapeutic and prognostic options, further collaborative research has to be pursued.
